# Improving preventive and health promotion care for children

**DOI:** 10.1186/s13584-018-0259-3

**Published:** 2018-10-01

**Authors:** Moira Inkelas, Frank Oberklaid

**Affiliations:** 1 Fielding School of Public Health, University of California, Los Angeles, 650 S Charles Young Drive, Los Angeles, CA 90024 USA; 2 Centre for Community Child Health, Murdoch Children’s Research Institute, Royal Children’s Hospital, Flemington Road, Parkville, VIC 3052 Australia

**Keywords:** Well child care, children’s primary care, Early childhood development

## Abstract

The emerging science in early childhood development challenges past paradigms of health care. There is consideration within the profession of paediatrics, and health care more broadly, of how to make systems of care more responsive to the developmental and social needs of young children and their families. Some countries have physician-centric models, either general physicians or paediatricians, while others rely on nurses. There is increasing recognition that the goal of any model should be parent-professional partnership that puts families at the center, elicits and responds to family needs, anticipates and supports families with developmental transitions, and fits within a seamless system of services and supports.

Chen Stein-Zamir and colleagues [1] offer an Israeli perspective on what health care in the early childhood period should look like. Their paper examines the roles and competencies of medical providers delivering well child care in Israel’s Maternal Child Health Clinics. As in previous inquiries into professional practice and parent preferences, this paper asks fundamental questions about the role of health professionals, especially physicians, in well care for young children (under age 6 years). The role of physicians, and health care professionals more broadly, vary by country so there is value in discussing these questions [2–4]. A more fundamental question to ask is the extent to which our existing health systems are designed to achieve good outcomes, in light of evolving evidence of the factors influencing early childhood development, and what organization of services best fits that evidence.

The development of physical, cognitive, and socioemotional capacities shape children’s life chances. The cost and intensity of intervention to mitigate problems increases with age, during childhood as well as in adulthood [5, 6]. Insight into how the science of early childhood development should guide models of supports and services [7] is leading to expanded focus on how to support families in the early years. Health professionals are well positioned to counsel parents to support children’s development, identify developmental concerns, identify family and social assets and risks that often affect development, and link families to early intervention and other community resources. For their part, families are eager to engage with professionals for information and support in how to foster optimal development [8–10]. Studies of parent preferences show that they prioritize and seek information and support according to their own needs [1, 8, 9]. The theme of parent questions about their child’s development and behaviour is consistent across studies. Beginning a conversation about these domains can open up a deeper discussion about pressures and challenges at a family level.

Kuo and colleagues [3, 4] offered a typology of preventive and health promotion care to enable comparisons of roles and personnel across countries. The typology of care includes monitoring development, planned anticipatory guidance (planned health education, generally established in a periodicity schedule that follows from American Academy of Pediatrics (AAP) guidelines), counselling for targeted topics, and follow-up and care coordination. In a study of selected Organisation for Economic Co-operation and Development (OECD) countries, Kuo et al. noted that most often nurses rather than physicians provide anticipatory guidance and parenting education, as well as problem-focused counselling for developmental concerns raised by parent or elicited by health professionals. Less often, general practitioners or paediatricians were responsible for such care. Countries with systems in which nurses or general practitioners provide problem-focused counselling also refer families to a paediatrician for specialized developmental services [4].

The AAP developed *Bright Futures* as a national health promotion and prevention initiative for children’s health [11]. *Bright Futures* includes evidence-based guidelines for periodicity and content recommendations; a schedule of planned encounters with recommended content for paediatric providers; educational materials for families; and professional development in health and developmental topics. The intent is to establish a consistent, evidence-driven schedule of content for families. *Bright Futures’* recommendations far exceed what can be accomplished in most health systems so the intent is for providers to adapt the guidelines based on the priorities for the families in their care.

Previous studies describe tensions between expectations and practice, particularly in preventive and health promotion care for younger children [3, 8, 12–15]. Primary care in the United States (U.S.) is not set up to accommodate the scope and depth of *Bright Futures*. Visit schedules were aligned initially with immunization periodicity rather than key developmental/behavioural milestones. Clinicians find there is insufficient time to address and elaborate on the full range of relevant issues at each visit, let alone respond adequately to parent concerns [14–16]. Models such as *Touchpoints* [17] and *Healthy Steps* [18, 19] emerged to respond to the early childhood science. Some features of these models are challenging to deliver in physician-centric systems when encounters are brief and episodic. The U.S. payment model does not support the dedicated child-development specialist within practices that the *Healthy Steps* approach calls for [12]. Additional challenges include having a narrow view of scope of medical practice, lack of other early childhood professionals within the workflow, and being siloed from other parts of the health-promoting system [16, 20–22]. There is a need to work across sectors and with other professionals to identify and address social issues [23, 24].

What should child health care offer, apart from addressing the common acute problems that children present with? Starting with the family’s problem/concern is central in rethinking models of care [14, 25]. The international health care improvement campaign centered on “what matters to you” [26] aligns with this foundation. A “think family” mindset [27] appreciates the importance of social context and recognizes the family as the greatest influence on early childhood development, especially in the first 1,000 days. This includes attention to family assets as well as concerns such as depression, and trauma [25, 28]. There is also growing appreciation of the need of health care providers to work as a team, with professionals from different disciplines, ideally in the same clinic [12]. This co-location is not always possible, so instead there is focus on the *system* of care, with close collaboration and coordination, seamless and efficient referral pathways, and reliable follow up [14, 15, 29].

There are examples in different countries of redesigning and standardizing well child care so that it covers all of these areas. The *Key Ages & Stages* framework [30] adopted in Victoria, Australia specifies developmental and anticipatory guidance content across a planned and scheduled series of visits. In the United Kingdom, the National Health Service *Preparation for Birth and Beyond* [31] describes antenatal content that augments the existing schedule of health visits in light of identified gaps and emerging science. Some systems need more infrastructure, especially where responsibility is scattered across systems. In the U.S., *Help Me Grow* [32] is a national system model that promotes cross-sector collaboration to build efficient and effective early childhood systems that mitigate the impact of adversity and support protective factors among families. In Israel, the *Goshen* model aims to increase the capability of child health care providers to address health and developmental needs of children [33, 34]. This educational transformation involves integrating developmental-behavioral paediatrics into continuing medical education and residency training while establishing fellowship training to prepare future leaders in community paediatrics [33]. Emerging approaches to care re-design and improvement across a system are also promising [35].

Few studies have been structured to answer the question of which roles within primary care are best met by which types of professionals. Features of different systems – in financing, organization, and nuances of care delivery – often confound the relationships that we observe. What is likely to matter most is the fulfilment of specific roles, with developmental expertise available and with parent-professional partnerships at the center. Co-located care, direct service, and consultation of a developmental specialist or occupational therapist to a primary care team are all possible mechanisms for organizing effective well child care. In practical terms, in some systems enhancing care means building capability primarily of physicians where in other systems, the focus is building capability of a nursing workforce. In nearly all systems, enhancing the care team in a place or virtually is a goal.

## Conclusion

Health care delivery often lags behind the science because of the challenge of translating knowledge into system reform and changes in practice. Translation often relies on continuing education that offers clinical content and protocols but does not support in re-designing workflow to accommodate the new way of practicing. Involvement of frontline providers in the re-design of services and practice is also important and not always emphasized. It is in this context that the contribution by Chen Stein-Zamir and colleagues is to be welcomed. In every country, including Israel, there needs to be a robust debate about how services are delivered to young children and their families – both the service delivery system and the practice of clinicians working in the system. The early childhood and life course research draw our attention to the opportunities for prevention and early intervention in the early years; to disregard this science and not to embrace the need for service reform is to do an injustice to children and their families, and has consequences for Israeli society in terms of social cohesion and missed economic opportunities.

## Abbreviations

AAP: American Academy of Pediatrics
OECD: Organisation for Economic Co-operation and Development
US: United States of America
